# Structural basis for bivalent binding and inhibition of SARS-CoV-2 infection by human potent neutralizing antibodies

**DOI:** 10.1038/s41422-021-00487-9

**Published:** 2021-03-17

**Authors:** Renhong Yan, Ruoke Wang, Bin Ju, Jinfang Yu, Yuanyuan Zhang, Nan Liu, Jia Wang, Qi Zhang, Peng Chen, Bing Zhou, Yaning Li, Yaping Shen, Shuyuan Zhang, Long Tian, Yingying Guo, Lu Xia, Xinyue Zhong, Lin Cheng, Xiangyang Ge, Juanjuan Zhao, Hong-Wei Wang, Xinquan Wang, Zheng Zhang, Linqi Zhang, Qiang Zhou

**Affiliations:** 1 Center for Infectious Disease Research, Westlake Laboratory of Life Sciences and Biomedicine, Key Laboratory of Structural Biology of Zhejiang Province, School of Life Sciences, Westlake University, 18 Shilongshan Road, Hangzhou, Zhejiang 310024 China; 2 Institute of Biology, Westlake Institute for Advanced Study, 18 Shilongshan Road, Hangzhou, Zhejiang 310024 China; 3 Comprehensive AIDS Research Center and Beijing Advanced Innovation Center for Structural Biology, School of Medicine, and Vanke School of Public Health, Tsinghua University, Beijing, 100084 China; 4 Tsinghua-Peking Joint Center for Life Sciences, Beijing, 100084 China; 5 Institute for Hepatology, National Clinical Research Center for Infectious Disease, Shenzhen Third People’s Hospital, Shenzhen, 518112 China; 6 The Second Affiliated Hospital, School of Medicine, Southern University of Science and Technology, Shenzhen, 518055 China; 7 The Ministry of Education Key Laboratory of Protein Science, Beijing Frontier Research Center for Biological Structure, Beijing, 100084 China; 8 Beijing Advanced Innovation Center for Structural Biology, Beijing Frontier Research Center for Biological Structure, Beijing, 100084 China; 9 Collaborative Innovation Center for Biotherapy, Tsinghua University, Beijing, 100084 China; 10 School of Life Sciences, Tsinghua University, Beijing, 100084 China; 11 Shenzhen Bay Laboratory, Shenzhen, Guangdong, 518055 China

**Keywords:** Cryoelectron microscopy, Mechanisms of disease

## Abstract

Neutralizing monoclonal antibodies (nAbs) to severe acute respiratory syndrome coronavirus 2 (SARS-CoV-2) represent promising candidates for clinical intervention against coronavirus disease 2019 (COVID-19). We isolated a large number of nAbs from SARS-CoV-2-infected individuals capable of disrupting proper interaction between the receptor binding domain (RBD) of the viral spike (S) protein and the receptor angiotensin converting enzyme 2 (ACE2). However, the structural basis for their potent neutralizing activity remains unclear. Here, we report cryo-EM structures of the ten most potent nAbs in their native full-length IgG-form or in both IgG-form and Fab-form bound to the trimeric S protein of SARS-CoV-2. The bivalent binding of the full-length IgG is found to associate with more RBDs in the “up” conformation than the monovalent binding of Fab, perhaps contributing to the enhanced neutralizing activity of IgG and triggering more shedding of the S1 subunit from the S protein. Comparison of a large number of nAbs identified common and unique structural features associated with their potent neutralizing activities. This work provides a structural basis for further understanding the mechanism of nAbs, especially through revealing the bivalent binding and its correlation with more potent neutralization and the shedding of S1 subunit.

## Introduction

The global pandemic of coronavirus disease 2019 (COVID-19) caused by the severe acute respiratory syndrome coronavirus 2 (SARS-CoV-2) is a serious threat to human health.^1,2^ SARS-CoV-2 is an enveloped, positive-stranded RNA virus, belonging to the beta-coronavirus genus that also includes SARS-CoV^3^ and the Middle Eastern respiratory syndrome coronavirus (MERS-CoV)^4^ that caused epidemic in 2003 and 2012, respectively. SARS-CoV-2 shares about 80% sequence identity with SARS-CoV, and both use angiotensin-converting enzyme 2 (ACE2) as their cellular receptor^5–9^ that is recognized and bound by the trimeric spike (S) protein.^10,11^ S protein distributes on the surface of the virion particles^12–14^ and is proteolytically cleaved into N-terminal S1 subunit and C-terminal S2 subunit during viral entry into target cells.^15^ S1 contains the N-terminal domain (NTD), the receptor binding domain (RBD), the subdomain 1 and 2 and is responsible for binding to receptor. S2 mediates the fusion of the viral and cellular membrane by undergoing a dramatic conformational change from the prefusion to the postfusion state^16^ accompanying with the shedding of S1. RBD, which directly binds to ACE2 receptor, is a major target for development of the therapeutic nAbs against COVID-19. The prefusion structure of S protein exhibits more dynamic conformational changes in S1 region, especially in RBD, which has two distinctive conformations, “up” and “down”.^10,11^ Only the “up” conformation of RBD can bind to the ACE2 receptor. Up to now, numerous nAbs against the S protein of SARS-CoV-2 have been reported.^17–38^ The complex structures of these nAbs with S protein were solved, most of which utilized the Fab-form of nAbs. It remains largely unknown how nAbs in their native bivalent form bind to and ever induce the conformational changes of the trimeric S protein.

To further explore the interactions between nAbs and S proteins, we solved cryo-electron microscopy (cryo-EM) structures of the S protein in complex with ten nAbs, in full-length IgG-form or in both IgG-form and Fab-form. Bivalent binding was revealed for the full-length form nAbs, which showed that the full-length form exhibits different binding mode and induces more RBDs to the “up” conformation than the Fab-form that is monovalent. The bivalent binding is superior in antiviral efficacy, and correlated with the improved shedding of the S1 subunit. Structural comparison of a large number of the complexes of nAbs with the S protein identified common and unique features associated with the potent neutralizing activities of these nAbs. Our results provide an important structural basis for further understanding the working mechanism of nAbs and are helpful for antiviral drug design and vaccine development.

## Results

### Potent nAbs isolated from the COVID-19 convalescent patients

To understand the molecular features of the interactions of neutralizing nAbs with the S protein, we characterized ten nAbs derived from COVID-19 convalescents with strong binding and neutralizing activities, and the capacity of competing with ACE2 for RBD binding. The binding affinity of these nAbs to RBD of SARS-CoV-2 measured by surface plasmon resonance (SPR) varied from 0.75 nM to 90.09 nM (Table 1; Supplementary information, Fig. S1), whereas the half maximal inhibitory concentration (IC_50_) of these nAbs ranged from 0.01 nM to 6.15 nM in the pseudovirus-based assay or from 0.03 nM to 5.95 nM in live SARS-CoV-2 virus-based assay (Table 1; Supplementary information, Fig. S1). All of these nAbs exhibited strong competition with ACE2 to bind RBD, indicating their neutralization mechanism (Table 1; Supplementary information, Fig. S1). The variable regions of the heavy chain of these nAbs belong to diverse gene families, paired with different families of light chains. The CDR3 length of the heavy chains and the light chains ranged from 9 to 22 amino acids and from 9 to 11 amino acids, respectively (Supplementary information, Table S1). The somatic hypermutation (SHM) of these nAbs were generally low and five of them contain no SHM for either heavy chain or light chain (Supplementary information, Table S1).

**Table 1:** Binding capacity, neutralizing and S1 shedding activity analysis of COVID-19 donor-derived neutralizing nAbs.

mAbs	IgG Binding to RBD		Live virus (nM)		Pseudovirus (nM)				Shedding at 120 min	
	Kd (nM)	competing with ACE2	IgG		IgG		Fab		IgG	Fab
			IC_50_	IC_80_	IC_50_	IC_80_	IC_50_	IC_80_		
P2B-1A10	50.77*	+++*	0.43*	2.04*	0.65*	4.96*	n.d.	n.d.	81.80%	n.a.
P5A-3A1	90.09	+++	4.48*	174.8*	6.15*	28.24*	n.a.	n.a.	77.20%	n.a.
P5A-1B8	1.09*	+++*	0.11*	0.57*	0.08*	0.33*	36.25	174.23	79.60%	25.14%
P5A-2G9	6.98*	+++*	0.08*	0.79*	0.11*	0.98*	n.d.	n.d.	84.90%	n.a.
P5A-1B6	1.01	+++	5.95*	39.42*	1.69*	9.15*	198.26	841.58	76.00%	n.a.
P2B-1A1	26.97	+++	1.48*	14.33*	4.60*	16.07*	n.a.	n.a.	−9.70%	n.a.
P5A-2G7	3.55*	+++*	1.21*	5.57*	0.03*	0.19*	195.49	1020.27	57.20%	15.51%
P5A-1B9	0.75*	+++*	0.03*	0.29*	0.01*	0.04*	9.12	35.37	43.80%	51.39%
P5A-2F11	5.33	+++	3.29*	46.28*	4.20*	12.93*	n.a.	n.a.	56.20%	n.a.
P5A-3C12	1.03*	+++*	1.76*	17.86*	0.66*	3.12*	138.74	500.33	54.50%	n.a.

Antibody binding to RBD was presented by Kd and competing with ACE2 where “+++” indicates > 80% competition. IC_50_ represents the half-maximal whereas IC_80_ the 80% inhibitory concentrations in the pseudovirus and live SARS-CoV-2 neutralization assay. Shedding at 120 min represents the S1 shedding abilities of IgG- or Fab-forms of antibodies, calculated by the reduction of the fluorescence at 120 min compared to that of 5 min incubation. * Published in Zhang, et al. Potent and protective IGHV3-53/3-66 public antibodies and their shared escape mutant on the spike of SARS-CoV-2 (submitted). n.d., not determined. n.a., not available.

Inducing shedding of the surface proteins of viruses is an important feature for nAbs.^39–41^ In our previous work, one potent neutralizing antibody P2C-1F11 was revealed to induce the shedding of S1 from the full-length S protein expressed on the cells into the supernatant over period of incubation.^42^ We decided to examine whether the nAbs uncovered here could induce the shedding of the S1 subunit of the S protein of SARS-CoV-2 by incubating antibodies with HEK293T cells expressing SARS-CoV-2 S protein over time. The results showed that after 120 min incubation, some nAbs such as P2B-1A10, P5A-1B8, P5A-2G9 and P5A-1B6, induced about 80% shedding of S1 subunit of the S protein. Other nAbs, such as P5A-2G7, P5A-1B9, P5A-2F11 and P5A-3C12, had only a weak shedding ability. P2B-1A1, however, exhibited almost no shedding ability, similar to the non-neutralizing antibody CR3022, suggesting variable capacity of these nAbs in inducing the shedding of the S1 subunit (Table 1; Supplementary information, Fig. S2a). Furthermore, shedding of S1 required full cleavage between S1 and S2 protein as the mutant S protein with GSAS substitution at the Furin cleavage site remained intact without obvious shedding (Supplementary information, Fig. S2b, c).

### The complex structures of the SARS-CoV-2 S trimer bound with nAbs

**Fig. 1:**
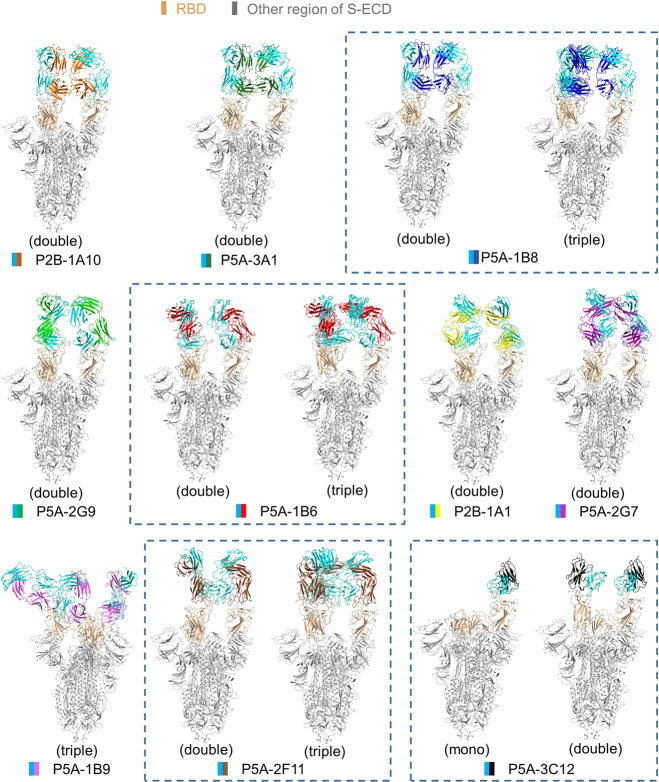
All solved structures of nAbs in complex with the S protein. The domain-colored models of all complex are shown. The structures containing different binding modes of the same nAb are boxed with blue dash line. The structures are labeled according to the number of RBD bound with nAb as mono (1 RBD), double (2 RBDs) or triple (3 RBDs) binding, respectively. The heavy chains of IgG are colored in cyan and the light chains of IgG are shown in the indicated colors.

To get deeper understanding of the working mechanism of these neutralizing antibodies, we determined the complex structures of the full-length IgG-form of these nAbs with the S protein of SARS-CoV-2 using single particle cryo-EM at overall resolution from 2.8 Å to 3.9 Å (Fig. 1; Supplementary information, Figs. S3–S7 and Table S2). The nAbs were incubated with the S protein at excessive molar ratio and the unbound nAbs were removed by gel filtration (Supplementary information, Fig. S3). For the region of RBD and bound nAbs, we performed focused refinement to obtain better resolution to build atomic models for nAb and allow detailed analysis (Supplementary information, Fig. S5), which was ranging from 3.0 Å to 4.2 Å for all nAbs except for P5A-3C12 that is 5.5 Å and only a docking model was used for it (Supplementary information, Table S2).

The structures of these S–IgG complexes can be classified into three different binding patterns (Fig. 1). In pattern 1 that includes P2B-1A10, P5A-3A1, P5A-2G9, P2B-1A1 and P5A-2G7, two “up” RBDs are bound with nAb. In pattern 2 that contains P5A-1B8, P5A-1B6 and P5A-2F11, two or three RBDs are in “up” conformation and bound with nAb, likely due to the potent binding capability of these antibodies to RBD (Table 1; Supplementary information, Fig. S1). Besides, one or two RBDs are in “up” conformation and bound with nAb for P5A-3C12 that belongs to the pattern 3. Compared to what we have observed with the S protein alone or with the S–ACE2 complex,^8^ the S–nAb complexes tend to have more RBD in the “up” conformation. P5A-1B9, the most potent neutralizing nAb as shown by the neutralization experiments against both live and pseudotyped virus in this work (Table 1; Supplementary information, Fig. S1), constitutes the pattern 4 that contains one “up” RBD and two “down” RBDs. All of three RBDs of the S protein in complex with P5A-1B9 are bound with nAb and share the same binding interface (Supplementary information, Fig. S8).

### The bivalent binding of nAbs

**Fig. 2:**
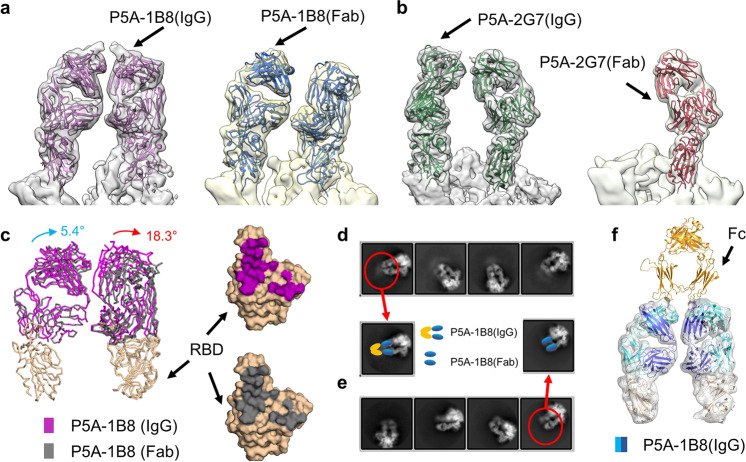
Bivalent binding analysis of nAbs. **a** Structural comparison between the S/P5A-1B8(IgG) complex and the S/P5A-1B8(Fab) complex. The cryo-EM maps docked with atomic models are shown. **b** Structural comparison between S/P5A-2G7(IgG) complex and the S/P5A-2G7(Fab) complex. **c** Comparison between P5A-1B8 (IgG) and P5A-1B8 (Fab) in complex with the S protein. The rotation of the variable region is larger on the right side (18.3°), comparing to the left side(5.4°). Whereas the IgG and the Fab complex have the same epitopes that are colored in purple and gray, respectively. **d**, **e** 2D classification of the S/P5A-1B8(IgG) complex and the S/P5A-1B8(Fab) complex re-centered at antibody, respectively. **f** The cryo-EM map of the S/P5A-1B8(IgG) complex can be docked with the Fc region of a full-length antibody model (PDB code: 5DK3). The Fc, heavy chain, and light chain of the antibody are colored in gold, blue and cyan, respectively.

It has been reported that the bivalent binding of antibodies can neutralize the virus more efficiently than Fab in some viruses such as rhinovirus and Dengue virus.^43,44^ To examine the structural difference of the complexes of the S protein with IgG and with Fab, we further solved the complex structures of the S protein with Fab region of P5A-1B8 or P5A-2G7 (Fig. 2; Supplementary information, Figs. S3, S4 and S9). In the S/P5A-1B8(IgG) complex, two or three RBDs are in “up” conformation and bound with nAb (Fig. 1), whereas only two RBDs are in “up” conformation and bound with nAb in the S/P5A-1B8(Fab) complex (Fig. 2a, right panel). Next, we compared the S/P5A-1B8(IgG) complex containing two “up” RBDs with the S/P5A-1B8(Fab) complex. The structural comparison showed that the two cryo-EM densities corresponding to the nAb in the S/P5A-1B8(IgG) complex are closer to each other than that in the S/P5A-1B8(Fab) complex (Fig. 2a). When the variable region of one of the Fab regions of the S/P5A-1B8(IgG) complex is superimposed with that of the S/P5A-1B8(Fab) complex, the constant region and other Fab undergo 5.4° and 18.3° rotation, respectively, whereas the interface remains unchanged (Fig. 2c, right panel). For P5A-2G7, only one Fab binds to one “up” RBD in the S/P5A-2G7(Fab) complex, whereas two “up” RBDs are bound with nAb in the S/P5A-2G7(IgG) complex (Fig. 2b). These results indicate different binding patterns between the IgG-form and the Fab-form nAbs.

We also re-centered the particles of the S/P5A-1B8(IgG) complex and the S/P5A-1B8(Fab) complex at the Fab region and performed the two-dimensional (2D) classification (Fig. 2d, e). The 2D class averages showed extra density near nAb of the S/P5A-1B8(IgG) complex, which might correspond to the Fc region of the P5A-1B8 (Fig. 2d) and was absent in the 2D class averages of the S/P5A-1B8(Fab) complex (Fig. 2e). Additionally, the Fab regions of an intact antibody molecule can be docked into the cryo-EM map of the S/P5A-1B8(IgG) complex (Fig. 2f). Taken together, these results support the bivalent binding exists for both P5A-1B8 and P5A-2G7.

### IgG-form nAbs shows advantage over Fab-form in neutralizing potency and shedding of S1 subunit

**Fig. 3:**
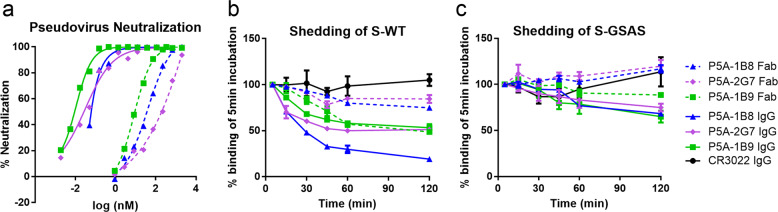
Neutralizing activity and shedding of S1 by IgG- and Fab-forms of nAbs. **a** Neutralizing activity against SARS-CoV-2 pseudovirus by P5A-1B8, P5A-1B9, and P5A-2G7 in IgG-forms (solid line) and Fab-forms (dotted line). Data were representative of at least two independent experiments. **b** Shedding of S1 over time measured using flow cytometry at 37 °C with 293T cell-surface expressed wild-type SARS-Cov-2 S protein. **c** Similar to **b**, with a mutant S protein containing GSAS substitution at S1/S2 cleavage site. Data were from three independent experiments, shown as means ± SEM.

To investigate the bivalent working mechanism more deeply, we compared the neutralizing potency and the ability to induce the shedding of S1 between the IgG- and the Fab-form of the most potent neutralizing antibody P5A-1B9, and two neutralizing antibodies P5A-1B8 and P5A-2G7 that have different binding patterns between IgG- and Fab-forms. As shown in Fig. 3a, the IgG-form nAbs exhibited higher neutralizing potency than the Fab-form of the same nAbs. The neutralizing activity measured by IC_50_ showed that the IgG-form was about 450- and 900-fold higher than that of Fab-form for P5A-1B8 (0.08 nM for IgG, 36.25 nM for Fab) and P5A-1B9 (0.01 nM for IgG, 9.12 nM for Fab). P5A-2G7 (0.03 nM for IgG, 195.49 nM for Fab) exhibited even more drastic differences of more than 6000-fold. Other IgG form nAbs in this work also showed higher neutralizing potency than the Fab form (Table 1; Supplementary information Fig. S2d). To compare the ability of inducing the shedding of S1 subunit between IgG- and Fab-forms, we incubated S protein-surface-expressed cells with IgG or Fab at saturated concentration and measured their binding over time by flow cytometry. As shown in Fig. 3b, the IgG of P5A-1B8 triggered S1 shedding most potently, resulting in about 80% shedding after incubating with cells for 120 min. However, the Fab-form of P5A-1B8 induced 25% shedding. IgG-form of P5A-2G7 showed mild shedding ability while Fab-form nearly lost the shedding ability. Both IgG- and Fab-form of P5A-1B9 showed similar mild shedding ability probably due to the long distances between Fab of P5A-1B9 in the complex with the S protein, which do not allow bivalent binding to two RBDs from one spike, suggesting the bivalent binding might contribute to the shedding capability (Fig.1; Supplementary information, Fig. S10). As a control and consistent with the expectation, S1 of the GSAS-containing mutant greatly reduced the shedding effect of IgG or Fab, whereas the negative control CR3022 failed to induce the shedding of S1 (Fig. 3c; Supplementary information, Fig. S2).

### Comparisons of antibody binding epitopes

**Fig. 4:**
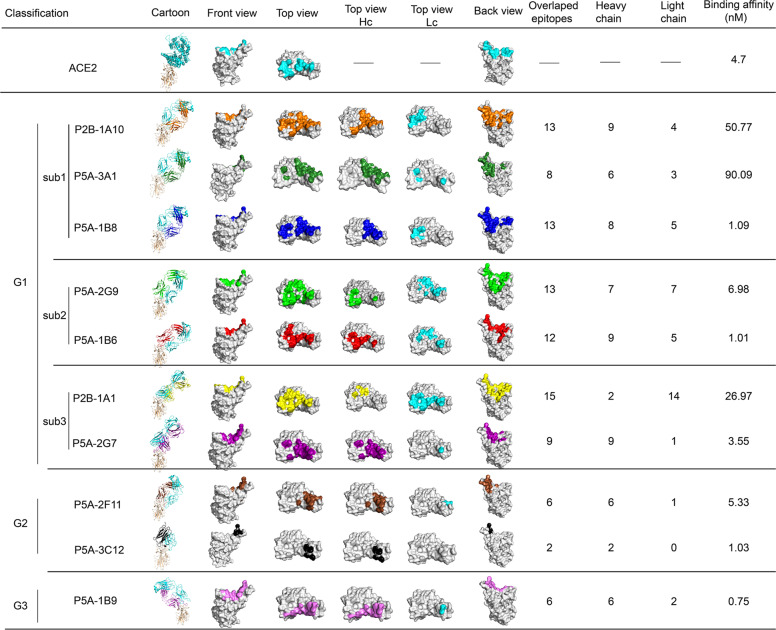
The classification and epitope of the 10 anti-SARS-CoV-2 nAbs. The 10 antibodies could be classified into three groups (G1, group 1; G2, group 2; G3, group 3). Group 1 antibodies can be further divided into three subgroups (sub1, sub2, sub3). The complexes of RBD with ACE2 or nAbs are shown as cartoon with RBD colored in wheat, the light chains of nAbs colored in cyan and the heavy chains of nAbs colored in different colors. For the epitope display, RBD is shown as gray surface in top, front and back views, with interface that binds to ACE2 colored in cyan and the epitopes of different nAbs shown in respective colors. For the top views, the epitopes corresponding to heavy chains are shown in respective colors (Top view Hc) and epitopes corresponding to light chains are shown in cyan (Top view Lc). Hc, heavy chain; Lc, light chain. The “Overlapped epitopes” column displays the residue number of nAb epitope that overlaps with ACE2-binding site. The “Heavy chain” and “Light chain” columns show the residue number of the epitope of the respective chain that overlaps with ACE2-binding site. The binding affinity to RBD of IgG antibodies was also shown.

A summary of the above cryo-EM structure determinations enables us to compare and classify the epitopes of these antibodies (Fig. 4; Supplementary information, Fig. S11). These ten neutralizing antibodies can be classified into three groups, considering the epitopes and approaching angles to RBD. The epitopes residues for the ten nAbs are summarized in Supplementary information, Table S3. The first group, including P2B-1A10, P5A-3A1, P5A-1B8, P5A-2G9, P5A-1B6, P2B-1A1 and P5A-2G7, has the largest overlap between the epitope and the ACE2-binding site on the RBD. Their epitope residues are distributed across the receptor binding motif (RBM), mainly in the cradle region. Among the 17 RBD residues involved in ACE2 binding, more than half (8 to 15) were recognized by the antibodies in group 1. The antibodies in group 1 also have similar contacting angles with RBD ranging from 39° to 52°, and can be further divided into three subgroups. The subgroup 1 consists of three antibodies P2B-1A10, P5A-3A1 and P5A-1B8, which use the same heavy chain IGHV3-53 V gene (Supplementary information, Table S1) and exhibit very similar positional arrangement. For antibodies in subgroup 1, the heavy chain plays a leading role. Among the 17 RBD residues involved in ACE2 binding, 6 to 9 were recognized by the heavy chains in subgroup 1 while 3 to 5 by light chains. They possessed the strongest shedding ability that are higher than 77.2% (Table 1). When RBD of the structures in first subgroup was aligned, the centroid distances of VH domains or VL domains are ranging from 1 Å to 2.3 Å or from 2.4 Å to 3.3 Å, respectively. The usage of the IGHV3-53/IGHV3-66 V genes have also been reported for other antibodies such as B38 and CB6.^21,23–26^

Subgroup 2 contains P5A-2G9 and P5A-1B6, both of which use heavy chain genes from IGHV 3 family, but not IGHV3-53 (IGHV3-33 for P5A-2G9 and IGHV3-30 for P5A-1B6, Supplementary information, Table S1). Subgroup 2 has a different VH and VL positional arrangement from the subgroup 1. Subgroup 3 contains P2B-1A1 and P5A-2G7, which use heavy chain gene from IGHV4 family (IGHV4-59 for P2B-1A1 and IGHV4-61 for P5A-2G7, Supplementary information, Table S1) and have a rotation around the longitudinal axis of the Fab compared with the antibodies in subgroup 1 and 2. The heavy chain of P5A-2G7 plays the leading role like subgroup 1, whereas the light chain of P2B-1A1 plays the leading role.

Group 2 consists of antibodies P5A-2F11 and P5A-3C12, which use heavy chain genes from IGHV1 and IGHV2 family (IGHV1-8 for P5A-2F11 and IGHV2-5 P5A-3C12, Supplementary information, Table S1), respectively. The epitopes for antibodies in group 2 are mainly located in the remote loops and less overlapped with ACE2-binding site, with only 6 or 2 overlapping residues for P5A-2F11 or P5A-3C12, respectively. The heavy chain of P5A-2F11 and P5A-3C12 plays the leading role.

The P5A-1B9, which uses heavy chain genes IGHV4-59 (Supplementary information, Table S1), alone forms group 3 among the ten neutralizing antibodies. The inter-molecular angle between P5A-1B9 and RBD is 52° anti-clockwise (Supplementary information, Fig. S11). Upon binding, P5A-1B9 approaches RBD from a direction different from those of the group 1 and 2 antibodies. The epitope for P5A-1B9 has 6 residues overlapping with ACE2-binding site, among which the heavy chain and light chain interacts with 6 and 2 residues, respectively.

## Discussion

In this work, we solved complex structures of the S protein with ten nAbs in full-length IgG-form or in both IgG-form and Fab-form. Structural analysis revealed the bivalent binding mode of nAbs against SARS-CoV-2. Our biochemical and cell-based experimental results showed that the full-length IgG nAbs have greater neutralization ability, and induce more S1 shedding than Fab (except P5A-1B9, for which IgG and Fab have similar shedding ability). Similar results were also obtained in another study.^27^ Moreover, there are more RBDs in “up” conformation in the structures of the S protein when complexed with full-length IgG than with Fab. The difference is likely attributed to the bivalent binding of IgG molecules that contain two Fab molecules. Such bivalent binding mode of full-length IgG nAbs has higher probability than Fab to exert stronger neutralization. Furthermore, the conformational change induced by the bivalent binding on the S protein is expected to translate to that on a virus surface, thereby disrupting the normal interaction between RBD and receptor ACE2.

In subgroup 1 of group 1, P5A-3A1 has similar positional arrangement to P5A-1B8, but much fewer overlapped residues with ACE2 than P5A-1B8, probably explaining the weaker binding affinity and neutralization ability of P5A-3A1 IgG (Kd 90.09 nM, IC_50_ 4.48 nM) than P5A-1B8 IgG (Kd 1.09 nM, IC_50_ 0.11 nM). Whereas for P5A-3A1 IgG, the bivalent binding might contribute to its neutralization ability. This might explain the large difference of neutralization potency between P5A-3A1 IgG and Fab.

Among the ten nAbs reported in this research, subgroup 1 and 2 of group 1 have a higher shedding ability (over 76 %), while subgroup 3 of group 1, group 2 and group 3 show no or weaker shedding ability (43.8%–57.2%). Group 1 has the largest overlap with ACE2 binding site, while the members in subgroup 3 have a rotation around the longitudinal axis of the Fab. The S1 shedding ability of the nAbs may be facilitated by the large overlap with ACE2 binding site and require special angle to bind with RBD. The most potent nAb P5A-1B9 only has mild shedding ability, whereas P5A-3A1 with relative weak neutralization ability possesses high shedding ability, suggesting the shedding ability contributed partially to the total neutralization ability.

The SARS-CoV and SARS-CoV-2 cross-reactive antibodies that show neutralizing activity mainly target to RBD, interfere with ACE2 binding and stimulate S1 dissociation,^40^ suggesting that the ability to induce the shedding of S1 is correlated with the potency of the neutralizing nAbs. The shedding of S1 might be facilitated by the RBD in “up” conformation that is stabilized by the binding of nAb or receptor. It is not clear whether the shedding of all three S1 subunits is required for the transition from the prefusion state to the postfusion state to catalyze the fusion of viral and cellular membrane. Our previous work indicates the receptor ACE2 exists as a dimer,^8^ of which each protomer of ACE2 dimer can be bound with one RBD from an S protein trimer and induces the bound RBD to the “up” conformation. It seems unlikely that one ACE2 dimer bivalently binds to a trimeric S protein simultaneously because of the steric constraint. To induce more than one RBDs in one S protein to up conformation, multiple copies of ACE2 are required, except when RBD can undergo a large rotation as suggested in a previous study.^45^

Many specific antibodies targeting S proteins are available in the Protein Data Bank (PDB) database now.^17–38,42^ We chose some representative antibodies and aligned them on the RBD of S protein (Supplementary information, Fig. S12). We divided these antibodies into four classes: Class I, their epitope residues are distributed across the RBM and compete the ACE2 binding, which mainly contains CB6 (PDB code:7C01), BD-604 (PDB code: 7CH4), S2E12 (PDB code: 7K4N), C105 (PDB code: 6XCN), C102 (PDB code: 7K8M), B38 (PDB code: 7BZ5) and P2C-1F11 (PDB code: 7CDI). Group 1 and group 2 of our antibodies belong to this class. Class II, their epitopes bind to RBD on the opposite side and partially overlapped with class I, which contains BD-368-2 (PDB code: 7CHH), CV07-270 (PDB code: 6XKP), S2H13 (PDB code: 7JV6), P2B-2F6 (PDB code: 7BWJ), S2M11 (PDB code: 7K43) and C002 (PDB code: 7K8T). The group 3 of our antibodies belong to this class. Class III, their epitopes are non-ACE2 competing site which contains H014 (PDB code: 7CAI), S2A4 (PDB code: 7JVC), S304 (PDB code: 7JW0) and CR3022 (PDB code: 6W41). Besides, there are some special antibodies that can compete ACE2 binding while bind to RBD with different patterns. We assigned these antibodies into class IV which contains S309 (PDB code: 6WPT), C110 (PDB code: 7K8V) and C135 (PDB code: 7K8Z). Among these nAbs, the group 3 nAb P5A-1B9 has the highest inhibitory activities against the cell infection of both pseudotyped and live SARS-CoV-2. Structure of the complex of P5A-1B9 with the S protein shows that it can bind to RBD in both “up” and “down” conformation, similar to the nAb BD-368-2 reported by another study,^25^ suggesting a common mechanism behind these nAbs of very high potency. Recently, the cocktail antibodies targeting different epitopes including the RBD region or NTD region of the S protein exhibit a magnified effect on neutralizing SARS-CoV-2 and also prevent the rapid mutational escape of host immune responses.^25,38,46,47^

## Materials and methods

### Antibody and Fab fragment production

Antibody production was conducted as previously described.^17^ Briefly, genes encoding the heavy and light chains of antibodies were transiently transfected into HEK 293 F cells using polyethylenimine (PEI) (Sigma). After 96 h, antibodies in the supernatant were collected and captured by Magnetic Protein A beads (Genscript). Bound antibodies were eluted and further purified by gel filtration chromatography using a Superdex 200 High Performance column (GE Healthcare). To produce Fab fragments, antibodies were cleaved using Protease Lys-C (Roche) with an IgG to Lys-C ratio of 4000:1 (w/w) in 10 mM EDTA, 100 mM Tris-HCl, pH 8.5 at 37 °C for approximately 12 h. Fc fragments were removed using Protein A Sepharose.

### Protein expression and purification

The extracellular domain (ECD) (1–1208 aa) was cloned into the pCAG vector (Invitrogen) with two proline substitutions at residues 986 and 987, a “GSAS” substitution at residues 682 to 685 and a C-terminal T4 fibritin trimerization motif followed by one Flag tag. The mutants were generated with a standard two-step PCR-based strategy.

The recombinant S-ECD protein (Genebank ID: QHD43416.1) was overexpressed using the HEK 293 F mammalian cells (Invitrogen) cultured in SMM 293T-II medium (Sino Biological Inc.) at 37 °C under 5% CO_2_ in a Multitron-Pro shaker (Infors, 130 rpm). When the cell density reached 2.0 × 10^6^ cells/mL, the plasmid was transiently transfected into the cells. To transfect one liter of cell culture, about 1.5 mg of the plasmid was premixed with 3 mg of PEIs (Polysciences) in 50 mL of fresh medium for 15 min before adding to cell culture. Cells were removed by centrifugation at 4000× *g* for 15 min after 60 h transfection. The secreted S-ECD proteins were purified by anti-FLAG M2 affinity resin (Sigma Aldrich). After loading two times, the anti-FLAG M2 resin was washed with the wash buffer containing 25 mM Tris (pH 8.0), 150 mM NaCl. The protein was eluted with the wash buffer plus 0.2 mg/mL flag peptide. The eluent was then concentrated and subjected to size-exclusion chromatography (Superose 6 Increase 10/300 GL, GE Healthcare) in buffer containing 25 mM Tris (pH 8.0), 150 mM NaCl. The peak fractions were collected and concentrated to incubate with Ab or Fab. The purified S-ECD was mixed with the Ab or Fab at a molar ratio of about 1:3.6 for one hour. Then the mixture was subjected to size-exclusion chromatography (Superose 6 Increase 10/300 GL, GE Healthcare) in buffer containing 25 mM Tris (pH 8.0), 150 mM NaCl. The peak fractions were collected for EM analysis.

SARS-CoV-2 RBD and the N-terminal peptidase domain of human ACE2 were expressed and purified by the same protocol as our previous work.^5^ An N-terminal gp67 signal peptide and a C-terminal 6× His tag were aligned with SARS-CoV-2 RBD (residues Arg319*–*Phe541) and inserted into the pFastBac-Dual vector (Invitrogen). The plasmid constructed was transformed into bacterial DH10Bac competent cells, and the bacmid extracted was then transfected into Sf9 cells using Cellfectin II Reagent (Invitrogen). Low-titer viruses were collected and amplified to generate high-titer virus stocks, which were used to infect Hi5 cells at the density of 2 × 10^6^ cells/mL. Supernatant of the cell culture containing secreted SARS-CoV-2 RBD was collected, concentrated and buffer-exchanged to HBS (10 mM HEPES, pH 7.2, 150 mM NaCl) 60 h after infection. SARS-CoV-2 RBD was captured by Ni-NTA resin (GE Healthcare), eluted with 500 mM imidazole in HBS buffer and then purified by gel filtration chromatography using a Superdex 200 column (GE Healthcare) pre-equilibrated with HBS buffer. Fractions containing SARS-CoV-2 RBD were collected. The N-terminal peptidase domain of human ACE2 (residues Ser19*–*Asp615) was expressed and purified by essentially the same protocol as the protocol used for SARS-CoV-2 RBD.

### Antibody binding kinetics and competition with receptor ACE2 measured by SPR

The binding kinetics and affinity of nAbs to SARS-CoV-2 RBD were analyzed by SPR (Biacore T200, GE Healthcare). Specifically, purified RBDs were covalently immobilized to a CM5 sensor chip via amine groups in 10 mM sodium acetate buffer (pH 5.0) for a final RU around 250. SPR assays were run at a flow rate of 30 μL/min in HEPES buffer. The sensograms were fit in a 1:1 binding model with BIA Evaluation software (GE Healthcare). To determine competition with the human ACE2 peptidase domain, SARS-CoV-2 RBD was immobilized to a CM5 sensor chip via amine group for a final RU around 250. Antibodies (1 μM) were injected onto the chip until binding steady-state was reached. ACE2 (2 μM) was then injected for 60 s. Blocking efficacy was determined by comparison of response units with and without prior antibody incubation.

### Neutralization of pseudotype and live virus

SARS-CoV-2 pseudoviruses were generated by co-transfecting HEK 293T cells (ATCC) with human immunodeficiency virus backbones expressing firefly luciferase (pNL43R E luciferase) and pcDNA3.1 (Invitrogen) expression vectors encoding S proteins. Viral supernatants were collected 48 h later. Pseudoviruses were incubated with serial dilutions of nAbs or Fab proteins at 37 °C for 1 h. Huh7 were then added in duplicate to the mixture. Antibody neutralization percentages were determined by measuring luciferase activity in relative light units (Bright-Glo Luciferase Assay Vector System, Promega Bioscience) 48 h after exposure to virus-antibody mixture using GraphPad Prism 7 (GraphPad Software Inc.). SARS-CoV-2 live virus focus reduction neutralization test (FRNT) was performed in a certified Biosafety level 3 laboratory as previously described. Neutralization assays against live SARS-CoV-2 were conducted using a clinical isolate (Beta/Shenzhen/SZTH-003/2020, EPI_ISL_406594 at GISAID) previously obtained from a nasopharyngeal swab of an infected patient. Serial dilutions of testing antibodies were mixed with 50 μL of SARS-CoV-2 (100 focus forming unit) in 96-well microwell plates and incubated at 37 °C for 1 h. Mixtures were then transferred to 96-well plates seeded with Vero E6 cells and allowed absorption for 1 h at 37 °C. Inoculums were then removed before adding the overlay media (100 μL MEM containing 1.6% Carboxymethylcellulose). The plates were then incubated at 37 °C for 24 h. Overlays were removed and then cells were fixed with 4% paraformaldehyde solution for 30 min, permeabilized with Perm/Wash buffer (BD Biosciences) containing 0.1% Triton X-100 for 10 min. Cells were incubated with rabbit anti-SARS-CoV-2 NP IgG (Sino Biological Inc.) for 1 h at room temperature before adding HRP-conjugated goat anti-rabbit IgG (H + L) antibody (TransGen Biotech, Beijing). The reactions were developed with KPL TrueBlue Peroxidase substrates (Seracare Life Sciences Inc.). The numbers of SARS-CoV-2 foci were calculated using an EliSpot reader (Cellular Technology Ltd.).

### Shedding of S1 from cell surface expressed SARS-CoV-2 S glycoprotein

Plasmids encoding SARS-CoV-2 S or mutant S containing GSAS, substituting RRAR at the junction between S1 and S2 to avoid digestion by Furin protease protein on the cell surface were transfected into HEK293T cells. Cells Samples were prepared in multiples for serial incubations at 37 °C for 120, 60, 45, 30, 15, or 5 min. Immediately after the allocated incubation time, antibody-stained cells were transferred to ice then thoroughly washed with ice-cold PBS and 2% FBS. Samples were then stained with anti-human IgG Fc PE (Biolegend 410718) for nAbs, or anti-human IgG (H + L) Alexa Flour 647 (ThermoFisher A21445) for Fab. After thorough washes with ice-cold PBS and 2% FBS, samples were resuspended and analyzed with FACS Calibur (BD Biosciences, USA) and FlowJo 10 software (FlowJo, USA). Binding at each of the allocated time points was determined by the MFI weighted by multiplying the number of positive cells in the selected gates and normalized in relative to that at the 5 min time point (Supplementary information, Fig. S13). The percentage of S1 shedding off at 120 min incubation were calculated by the different percentage of antibody binding between 120 min and 5 min incubation. Statistic difference of shedding ability of 10 nAbs between S-WT and S-GSAS was analyzed using Wilcoxon matched-paires *t-*test by GraphPad Prism 7.01.

### Cryo-EM sample preparation

The peak fractions of complex were concentrated to about 1.5 mg/mL and applied to the grids. Aliquots (3.3 μL) of the protein complex were placed on glow-discharged holey carbon grids (Quantifoil Au R1.2/1.3). The grids were blotted for 2.5 s or 3.0 s and flash-frozen in liquid ethane cooled by liquid nitrogen with Vitrobot (Mark IV, Thermo Scientific). The cryo-EM samples were transferred to a Titan Krios operating at 300 kV equipped with Gatan K3 detector and GIF Quantum energy filter. Movie stacks were automatically collected using AutoEMation,^48^ with a slit width of 20 eV on the energy filter and a defocus range from *–*1.2 µm to *–*2.2 µm in super-resolution mode at a nominal magnification of 81,000×. Each stack was exposed for 2.56 s with an exposure time of 0.08 s per frame, resulting in a total of 32 frames per stack. The total dose rate was approximately 50 e^−^/Å^2^ for each stack. The stacks were motion corrected with MotionCor2^49^ and binned 2-fold, resulting in a pixel size of 1.087 Å/pixel. Meanwhile, dose weighting was performed.^50^ The defocus values were estimated with Gctf.^51^

### Data processing

Particles were automatically picked using Relion 3.0.6^52–55^ from manually selected micrographs. After 2D classification with Relion, good particles were selected and subject to two cycles of heterogeneous refinement without symmetry using cryoSPARC.^56 ^The good particles were selected and subjected to non-uniform refinement (beta) without symmetry, resulting in the three-dimensional (3D) reconstruction for the whole structure, which was further subject to 3D auto-refinement and post-processing with Relion. For interface between S protein of SARS-CoV-2 and nAb, the dataset was subject to focused refinement with adapted mask on the region of the RBD–nAb sub-complex to improve the map quality. The dataset was re-centered on the interface between RBD and nAb and re-extracted. The dataset of multiple RBD–nAb sub-complexes in a S/nAb complex were combined if possible and necessary. The re-extracted dataset was 3D classified with Relion focused on RBD–nAb sub-complex. Then the good particles were selected and subject to focused refinement with Relion, resulting in the 3D reconstruction of better quality on RBD–nAb sub-complex.

The resolution was estimated with the gold-standard Fourier shell correlation 0.143 criterion^57^ with high-resolution noise substitution.^58^ Refer to Supplementary information, Figs. S3, S4 and Table S2 for details of data collection and processing.

### Model building and structure refinement

For model building of the complex of S-ECD of SARS-CoV-2 with nAb, a model was first obtained for the nAb with Chainsaw^59^ using a template (PDB ID: 7C2L). Then, the models of S-ECD (PDB ID: 7C2L) and the nAb were molecular dynamics flexible fitted^60^ into the whole cryo-EM map of the complex and the focused-refined cryo-EM map of the RBD–nAb sub-complex, respectively, which were merged and further manually adjusted with Coot.^61^ Each residue of the complex of S-ECD of SARS-CoV-2 with nAb was manually checked with the chemical properties taken into consideration during model building. Structural refinement was performed in Phenix^62^ with secondary structure and geometry restraints to prevent overfitting. To monitor the potential overfitting, the model was refined against one of the two independent half maps from the gold-standard 3D refinement approach. Then, the refined model was tested against the other map. Statistics associated with data collection, 3D reconstruction and model building were summarized in Supplementary information, Table S2.

## Supplementary information

Supplementary information, Fig. S1

Supplementary information, Fig. S2

Supplementary information, Fig. S3

Supplementary information, Fig. S4

Supplementary information, Fig. S5

Supplementary information, Fig. S6

Supplementary information, Fig. S7

Supplementary information, Fig. S8

Supplementary information, Fig. S9

Supplementary information, Fig. S10

Supplementary information, Fig. S11

Supplementary information, Fig. S12

Supplementary information, Fig. S13

Supplementary information, Table S1

Supplementary information, Table S2

Supplementary information, Table S3

## Data Availability

Atomic coordinates and cryo EM density maps of the nAbs in complex with S protein have been deposited to the Protein Data Bank (http://www.rcsb.org) and the Electron Microscopy Data Bank (https://www.ebi.ac.uk/pdbe/emdb/), respectively. Please refer to Supplementary information, Table S2 for the PDB and EMDB codes. Correspondence and requests for materials should be addressed to zhangzheng1975@aliyun.com; zhanglinqi@tsinghua.edu.cn; zhouqiang@westlake.edu.cn.
