# Supplementary material for: Structural basis for bivalent binding and inhibition of SARS-CoV-2 infection by human potent neutralizing antibodies
Source: Cell Res. 2021 Mar 17;31(5):517–25. doi: 10.1038/s41422-021-00487-9 (PMC7966918; doi:10.1038/s41422-021-00487-9)
Supplement: Supplementary file 1 — Supplementary information, Fig. S1 [file 41422_2021_487_MOESM1_ESM.pdf]

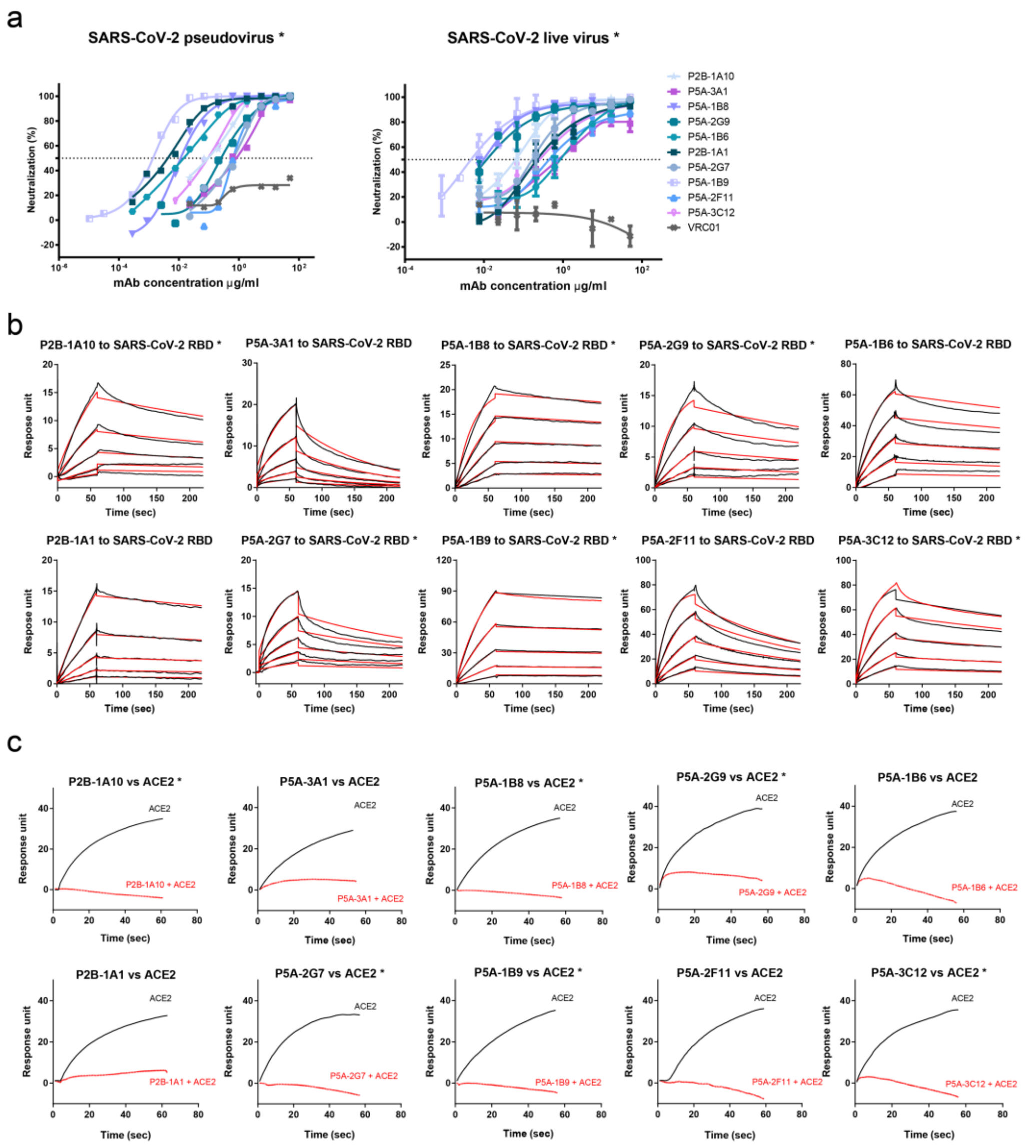

Supplementary information, Fig. S1 | Neutralizing activity, binding kinetics, and ACE2 competition of COVID-19 donor-derived nAbs.

a Neutralizing activities of the antibodies against SARS-CoV-2 pseudovirus and live virus. **b** binding kinetics of antibodies with soluble SARS-CoV-2 RBD measured by Surface Plasmon Resonance. The black lines indicate the experimentally derived curves while the red lines represent fitted curves by 1:1 binding fitness model. **c** binding patterns of ACE2 receptor protein to SARS-Cov-2 RBD with (red curve) or without (black curve) prior injection and saturation with each testing antibody.

* Published in the reference (Zhang, et al. Public neutralizing antibodies elicited by SARS-CoV-2 infection. submitted).
