# Supplementary material for: Structural basis for bivalent binding and inhibition of SARS-CoV-2 infection by human potent neutralizing antibodies
Source: Cell Res. 2021 Mar 17;31(5):517–25. doi: 10.1038/s41422-021-00487-9 (PMC7966918; doi:10.1038/s41422-021-00487-9)
Supplement: Supplementary file 2 — Supplementary information, Fig. S2 [file 41422_2021_487_MOESM2_ESM.pdf]

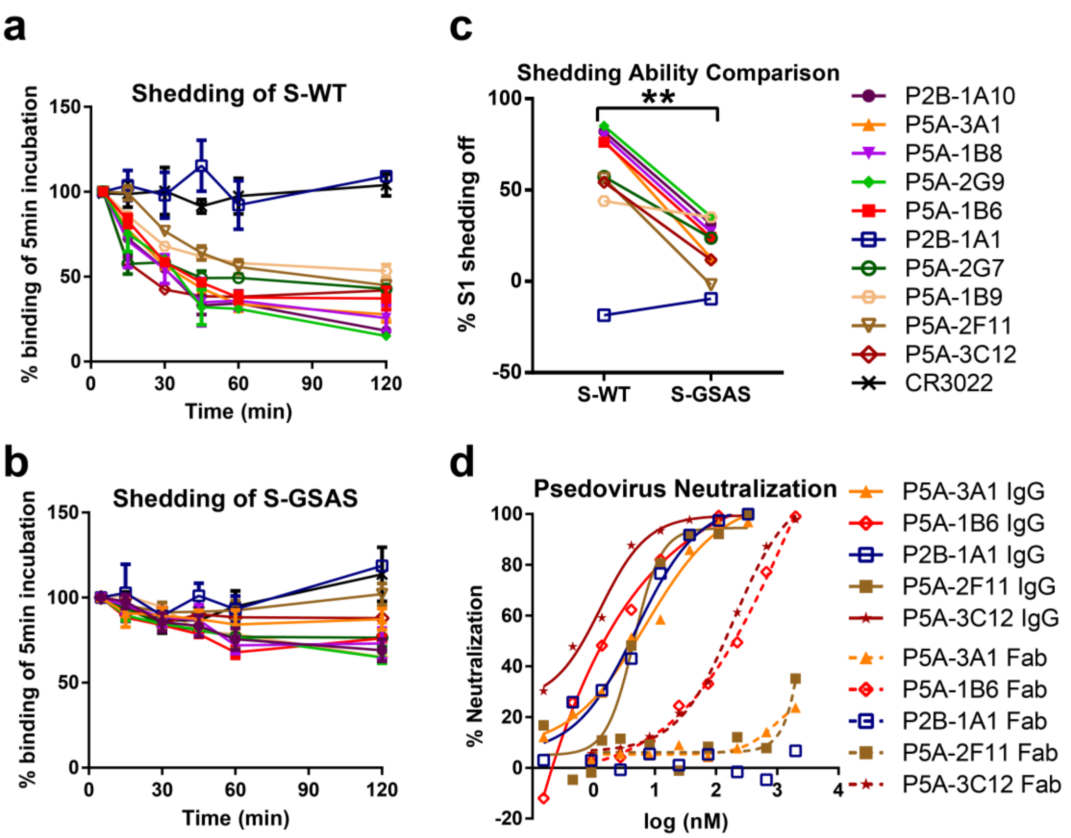

Supplementary information, Fig. S2 | The neutralization and shedding property of different antibodies.

Shedding of S1 over time was measured by flow cytometry at 37°C with 293T cell-surface expressed (**a**) wildtype SARS-CoV-2 spike or (**b**) a mutant spike containing GSAS substitution at S1/S2 cleavage motif. The percentage of cells at each allocated time point was determined by the MFI weighted by multiplying the number of positive cells in the selected gates and normalized in relative to the 5 min time point. P2B-2F6 and CR3022 was used as negative control. Data of **a** were from at least two independent experiments. Data of **a** and **b** were from three independent experiments. Values are indicated as mean±SEM. **c** statistic difference of the percentage of S1 shedding off at 120 min incubation by the 10 nAbs between S-WT and S-GSAS spike were analyzed using paired t test. ** P value<0.05. **d** Neutralizing activity against SARS-CoV-2 pseudovirus by rest IgG forms (solid line) and Fab forms (dotted line).
