# Supplementary material for: Structural basis for bivalent binding and inhibition of SARS-CoV-2 infection by human potent neutralizing antibodies
Source: Cell Res. 2021 Mar 17;31(5):517–25. doi: 10.1038/s41422-021-00487-9 (PMC7966918; doi:10.1038/s41422-021-00487-9)
Supplement: Supplementary file 3 — Supplementary information, Fig. S3 [file 41422_2021_487_MOESM3_ESM.pdf]

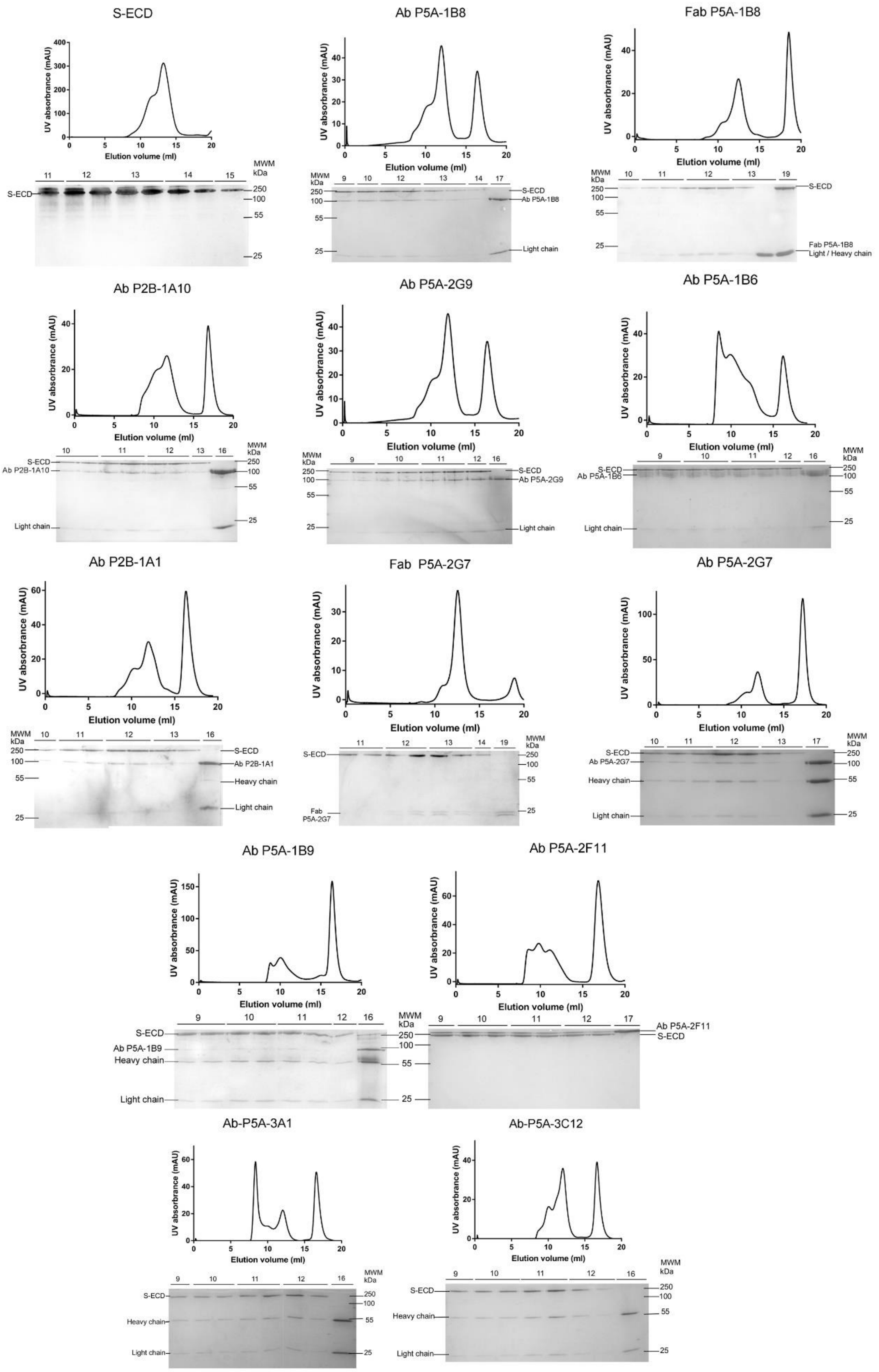
S-ECD
Ab P5A-1B8
Fab P5A-1B8
Ab P2B-1A10
Ab P5A-2G9
Ab P5A-1B6
Ab P2B-1A1
Fab P5A-2G7
Ab P5A-2G7
Ab P5A-1B9
Ab P5A-2F11
Ab-P5A-3A1
Ab-P5A-3C12
UV absorbance (mAU)
Elution volume (ml)
MWM
kDa
250
100
55
25
S-ECD
Ab P5A-1B8
Light chain
Fab P5A-1B8
Light / Heavy chain
Ab P2B-1A10
Ab P5A-2G9
Ab P5A-1B6
Ab P2B-1A1
Heavy chain
Fab
P5A-2G7
Ab P5A-2G7
Ab P5A-1B9
Ab P5A-2F11

Supplementary information, Fig. S3 | Representative SEC purification profile of the S-ECD of SARS-CoV-2 in complex with all kinds of nAbs.
