# Supplementary material for: Structural basis for bivalent binding and inhibition of SARS-CoV-2 infection by human potent neutralizing antibodies
Source: Cell Res. 2021 Mar 17;31(5):517–25. doi: 10.1038/s41422-021-00487-9 (PMC7966918; doi:10.1038/s41422-021-00487-9)
Supplement: Supplementary file 4 — Supplementary information, Fig. S4 [file 41422_2021_487_MOESM4_ESM.pdf]

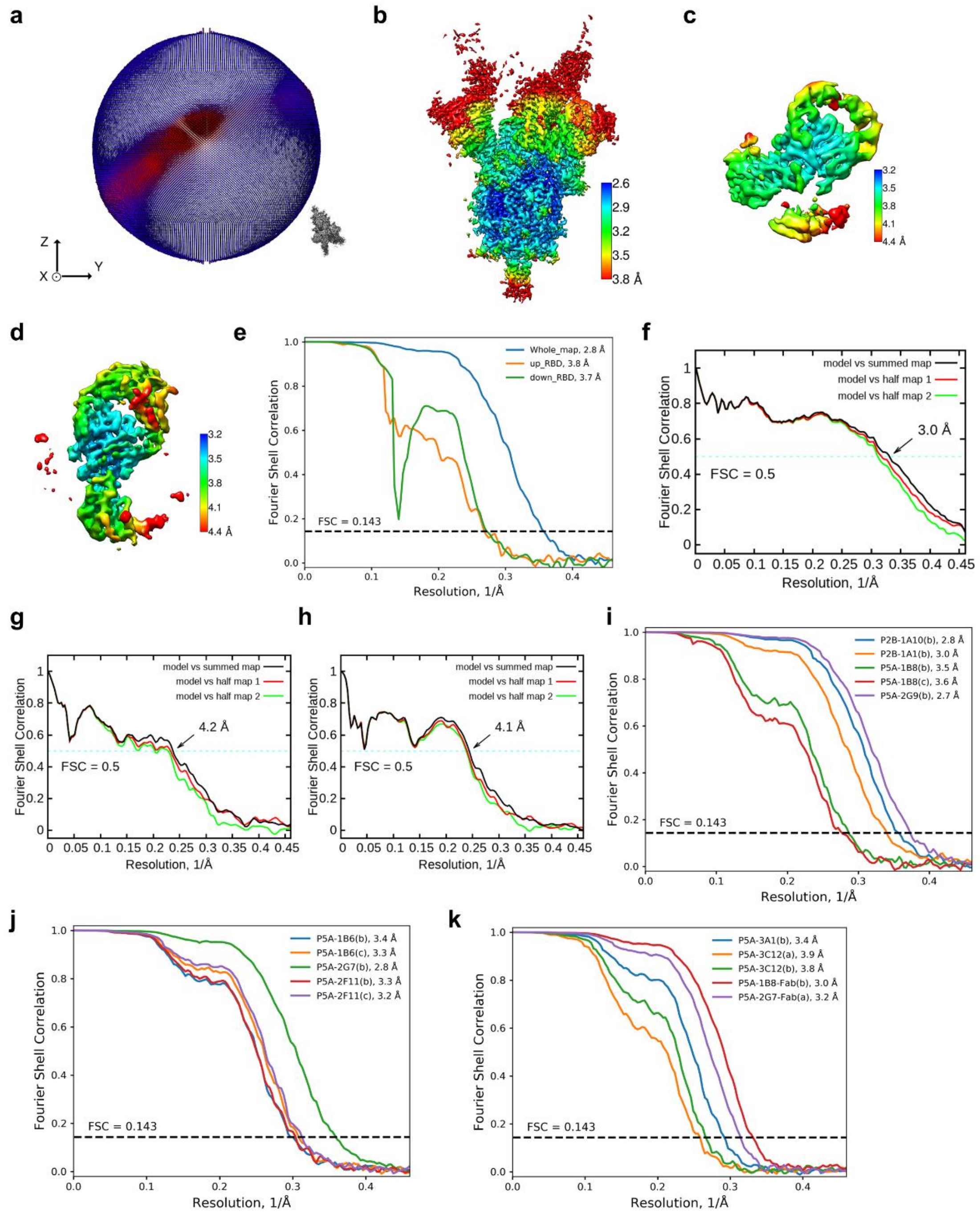

Supplementary information, Fig. S4 | Representative cryo-EM analysis of S-ECD in complex with nAbs.

a, Euler angle distribution in the final 3D reconstruction of S-ECD of SARS-CoV-2 bound with P5A-1B9 complex. **b-d** Local resolution map for the 3D reconstruction of overall structure, “down” RBD-P5A-1B9 sub-complex and “up” RBD-P5A-1B9 sub-complex, respectively. **e** FSC curve of the overall structure (blue), “up” RBD-P5A-1B9 sub-complex (orange) and “down” RBD-P5A-1B9 sub-complex (green). **f** FSC curve of the refined model of SARS-CoV-2 bound with P5A-1B9 complex versus the

overall structure that it is refined against (black); of the model refined against the first half map versus the same map (red); and of the model refined against the first half map versus the second half map (green). The small difference between the red and green curves indicates that the refinement of the atomic coordinates did not suffer from overfitting. **g** and **h** FSC curve of the refined model of “down” RBD-P5A-1B9 sub-complex and “up” RBD-P5A-1B9 sub-complex, which is same to the **f**. **i-k** Gold standard FSC curve of the overall structure for all kinds of nAb bound with S-ECD, respectively. (a), mono; (b) double;(c), triple.
