# Supplementary material for: Structural basis for bivalent binding and inhibition of SARS-CoV-2 infection by human potent neutralizing antibodies
Source: Cell Res. 2021 Mar 17;31(5):517–25. doi: 10.1038/s41422-021-00487-9 (PMC7966918; doi:10.1038/s41422-021-00487-9)
Supplement: Supplementary file 5 — Supplementary information, Fig. S5 [file 41422_2021_487_MOESM5_ESM.pdf]

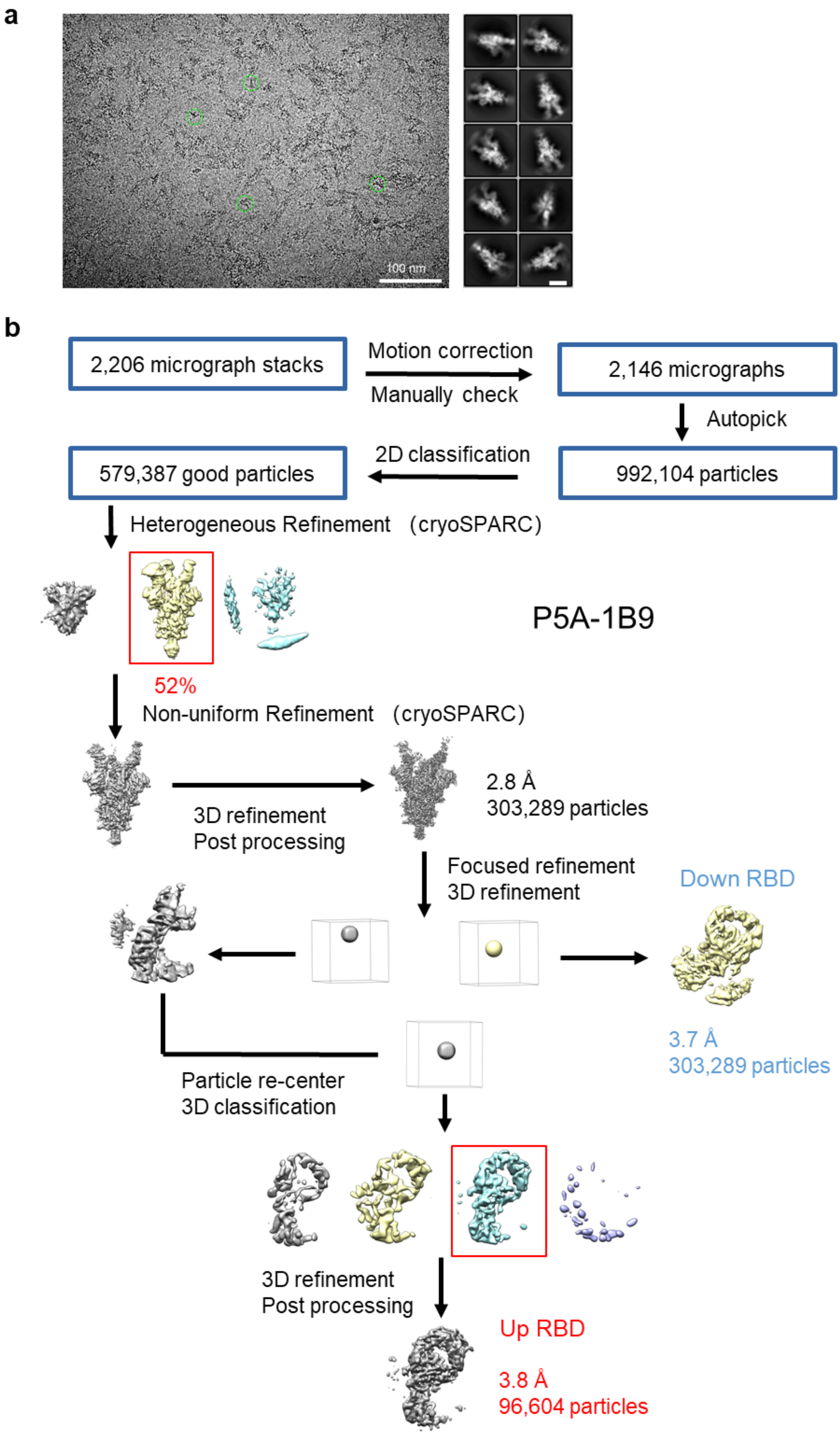
a
100 nm
b
2,206 micrograph stacks
Motion correction
Manually check
2,146 micrographs
Autopick
992,104 particles
2D classification
579,387 good particles
Heterogeneous Refinement (cryoSPARC)
P5A-1B9
52%
Non-uniform Refinement (cryoSPARC)
3D refinement
Post processing
2.8 Å
303,289 particles
Focused refinement
3D refinement
Down RBD
3.7 Å
303,289 particles
Particle re-center
3D classification
3D refinement
Post processing
Up RBD
3.8 Å
96,604 particles

Supplementary information, Fig. S5 | Representative cryo-EM image and flowchart for cryo-EM data processing of S-ECD in complex with P5A-1B9. a Representative electron micrograph and 2D class averages of cryo-EM particle images. The scale bar in 2D class averages represent 10 nm. **b** Please refer to the 'Data Processing' in Methods section for details.
