# Supplementary material for: Structural basis for bivalent binding and inhibition of SARS-CoV-2 infection by human potent neutralizing antibodies
Source: Cell Res. 2021 Mar 17;31(5):517–25. doi: 10.1038/s41422-021-00487-9 (PMC7966918; doi:10.1038/s41422-021-00487-9)
Supplement: Supplementary file 6 — Supplementary information, Fig. S6 [file 41422_2021_487_MOESM6_ESM.pdf]

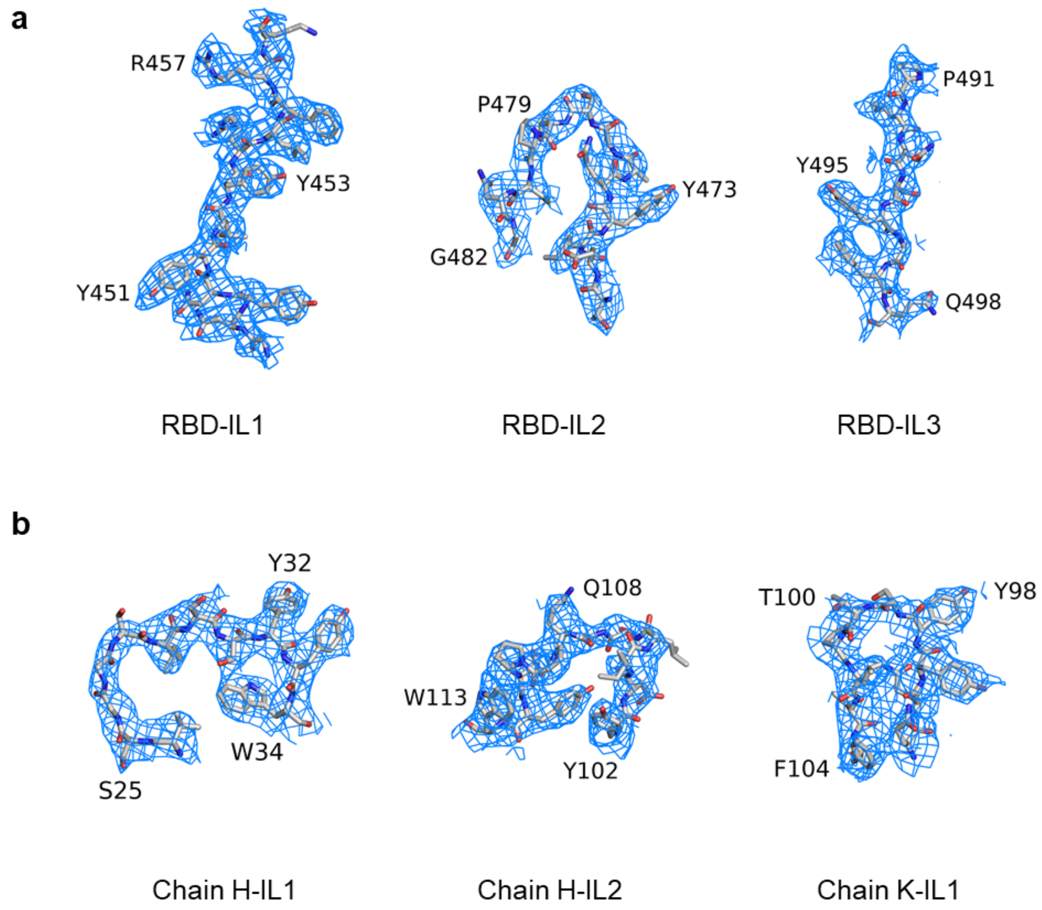

Supplementary information, Fig. S6 | Representative cryo-EM density maps.

a Cryo-EM density map for RBD of S-ECD in complex with P5A-1B9 shown at threshold of 8 σ. **b** Cryo-EM density map for nAb of S-ECD in complex with P5A-1B9 shown at threshold of 8 σ. IL, interface loop.
