# Supplementary material for: Structural basis for bivalent binding and inhibition of SARS-CoV-2 infection by human potent neutralizing antibodies
Source: Cell Res. 2021 Mar 17;31(5):517–25. doi: 10.1038/s41422-021-00487-9 (PMC7966918; doi:10.1038/s41422-021-00487-9)
Supplement: Supplementary file 8 — Supplementary information, Fig. S8 [file 41422_2021_487_MOESM8_ESM.pdf]

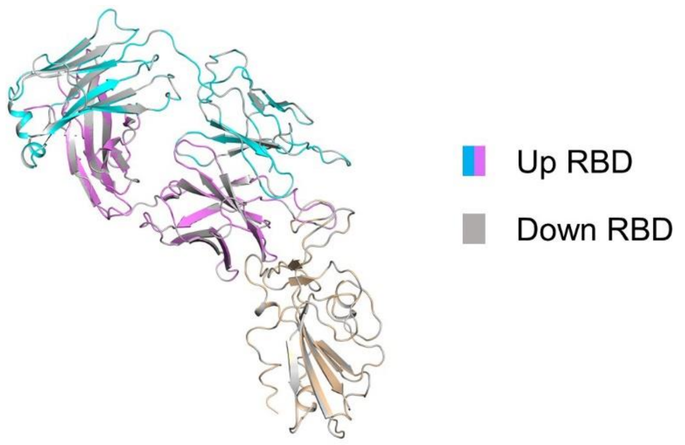

Supplementary information, Fig. S8 | Structural alignment of RBD-P5A-1B9 sub-complex.

Superposition of the structures of P5A-1B9 bound to “up” and “down” RBD indicates there is no difference between two structures. For clarity, only antibody and RBD are shown.
