# Supplementary material for: Structural basis for bivalent binding and inhibition of SARS-CoV-2 infection by human potent neutralizing antibodies
Source: Cell Res. 2021 Mar 17;31(5):517–25. doi: 10.1038/s41422-021-00487-9 (PMC7966918; doi:10.1038/s41422-021-00487-9)
Supplement: Supplementary file 9 — Supplementary information, Fig. S9 [file 41422_2021_487_MOESM9_ESM.pdf]

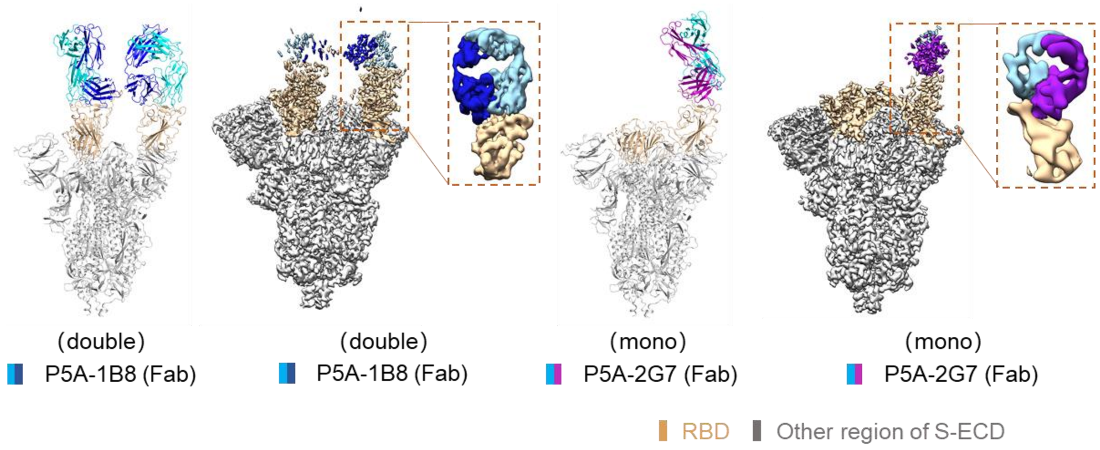

Supplementary information, Fig. S9 | Cryo-EM models and maps of S-ECD in complex with Fab.

The domain-colored models and cryo-EM maps of all complexes are shown here.
