# Supplementary material for: Structural basis for bivalent binding and inhibition of SARS-CoV-2 infection by human potent neutralizing antibodies
Source: Cell Res. 2021 Mar 17;31(5):517–25. doi: 10.1038/s41422-021-00487-9 (PMC7966918; doi:10.1038/s41422-021-00487-9)
Supplement: Supplementary file 10 — Supplementary information, Fig. S10 [file 41422_2021_487_MOESM10_ESM.pdf]

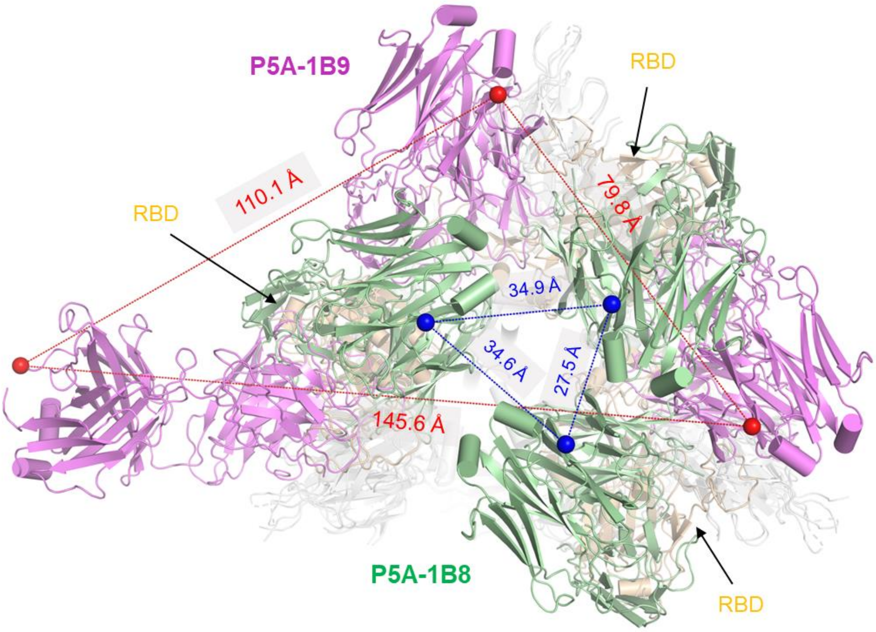

Supplementary information, Fig. S10 | The distance comparison between bound Fabs for P5A-1B8 and P5A-1B9.

The terminal distance for P5A-1B9 is larger than P5A-1B8 and that would not allow for a bivalent binding.
