# Supplementary material for: Structural basis for bivalent binding and inhibition of SARS-CoV-2 infection by human potent neutralizing antibodies
Source: Cell Res. 2021 Mar 17;31(5):517–25. doi: 10.1038/s41422-021-00487-9 (PMC7966918; doi:10.1038/s41422-021-00487-9)
Supplement: Supplementary file 11 — Supplementary information, Fig. S11 [file 41422_2021_487_MOESM11_ESM.pdf]

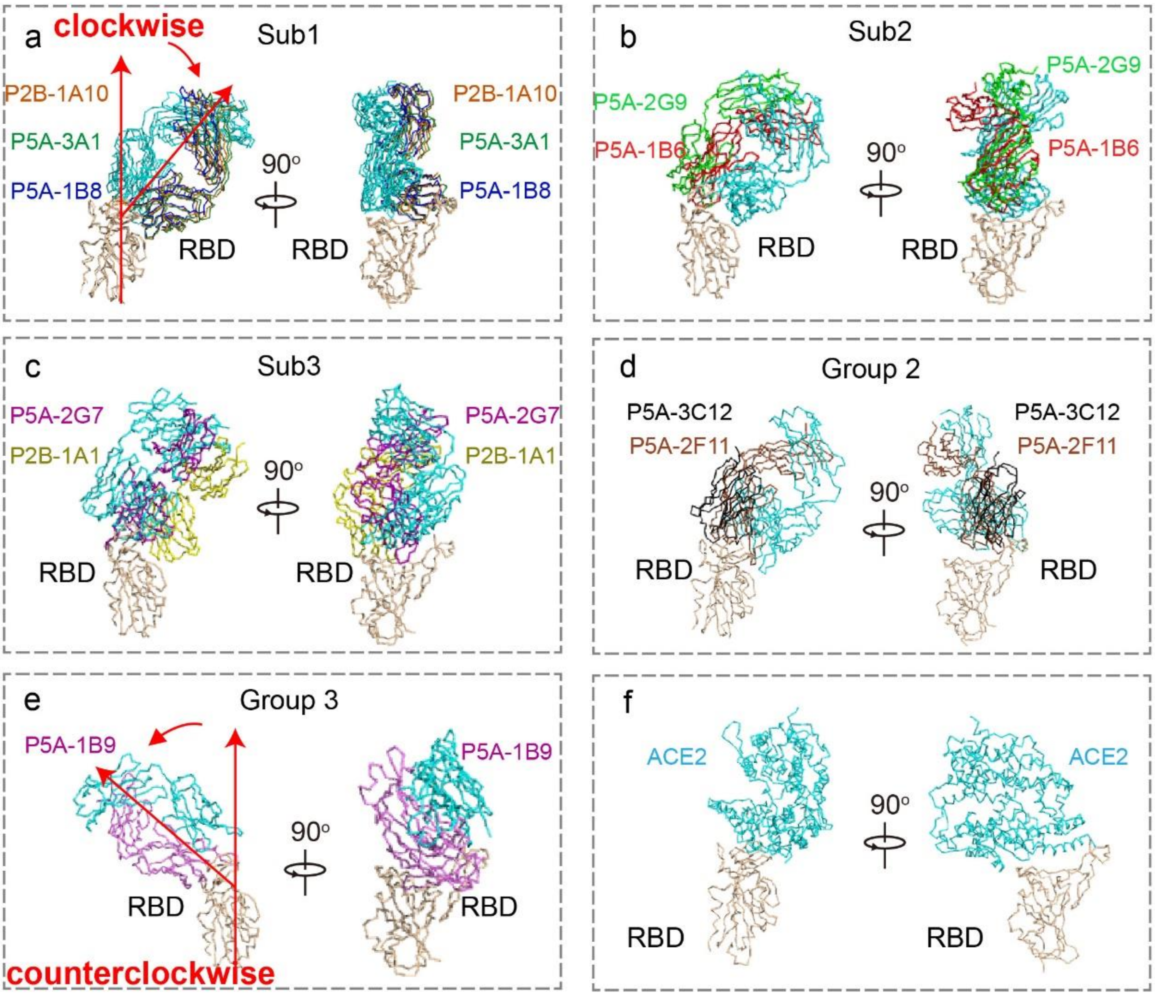


**Fig. S11 | Classification and exhibition of the 10 antibodies and ACE2.**

All RBD-nAb complexes and RBD-ACE2 are shown as ribbon mode in two vertical directions. The color mode of our 10 antibodies and ACE2 are the same as Fig 4. The vertical line is the first axis and the second axis is bisector of antibody. The angle of RBD and nAbs was calculated using protractor. **a** Antibodies of subgroup 1. Subgroup 1 consists of 3 antibodies P2B-1A10, P5A-3A1 and P5A-1B8. **b** Antibodies of subgroup 2. Subgroup 2 consists of 2 antibodies P5A-2G9 and P5A-1B6. **c** Antibodies of subgroup 3. Subgroup 3 consists of 2 antibodies P2B-1A1 and P5A-2G7. **d** Antibody of group 2. Group 2 consist of 2 antibodies P5A-2F11 and P5A-3C12. **e** Antibodies of group 3. Group 3 consists of 1 antibody P5A-1B9. **f** RBD-ACE2 complex in the same direction with other RBD-nAb complexes.
