# Supplementary material for: Structural basis for bivalent binding and inhibition of SARS-CoV-2 infection by human potent neutralizing antibodies
Source: Cell Res. 2021 Mar 17;31(5):517–25. doi: 10.1038/s41422-021-00487-9 (PMC7966918; doi:10.1038/s41422-021-00487-9)
Supplement: Supplementary file 12 — Supplementary information, Fig. S12 [file 41422_2021_487_MOESM12_ESM.pdf]

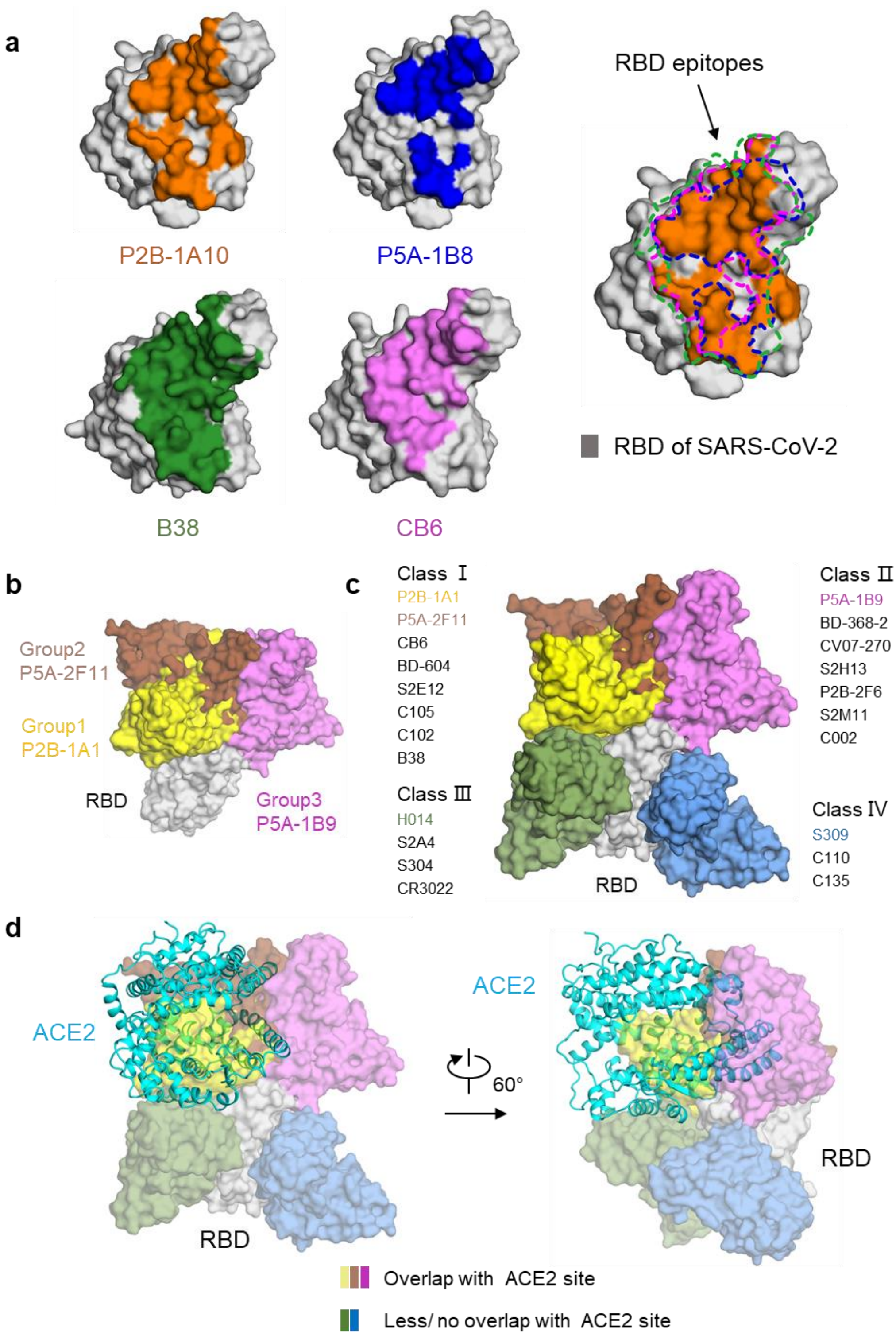

Supplementary information, Fig. S12 | Comparison and classification of nAbs. **a** Here are shown the RBD epitopes of nAbs from IGHV3-53 V genes such as P2B-1A10 (orange), P5A-1B8 (blue) and other reported antibodies such as B38 (green) and CB6 (violet). Right panel is the superposition of RBD epitopes of nAbs. **b**

Structural comparison of epitopes of nAbs from group 1, group 2 and group 3 bound to RBD, which show the representative nAbs such as P2B-1A1 (yellow), P5A-2F11 (brown) and P5A-1B9 (violet), respectively. **c** Structural classification of some representative nAbs from each class binding its RBD epitope. The typical antibodies of four classes are shown as P2B-1A1 (yellow) and P5A-2F11 (brown) of class 1, P5A-1B9 (violet) of class 2, H014 (green) of class 3 and S309 (blue) of class 4. **d** Structural classification between nAbs from four classes and ACE2.There are overlaps between class1 or class2 and ACE2, while there are nearly no overlaps between class3 or class4 and ACE2.
