# Supplementary figures and images for: Structural basis for bivalent binding and inhibition of SARS-CoV-2 infection by human potent neutralizing antibodies
Source: Cell Res. 2021 Mar 17;31(5):517–25. doi: 10.1038/s41422-021-00487-9 (PMC7966918; doi:10.1038/s41422-021-00487-9)

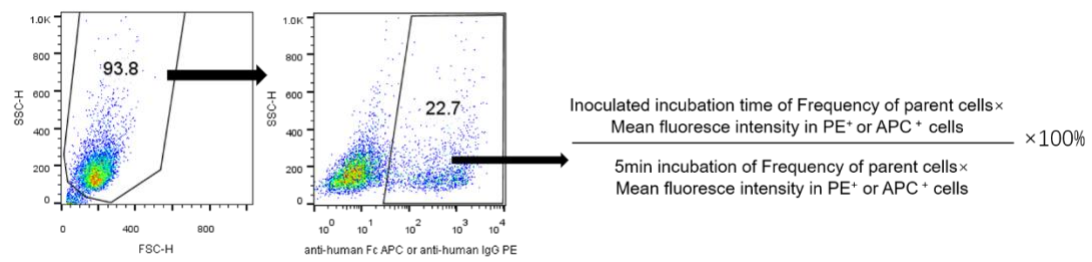

**Supplementary information, Fig. S13 | Gating strategies of S1 shedding assay.**

Supplement: Supplementary file 13 — Supplementary information, Fig. S13 [file 41422_2021_487_MOESM13_ESM.pdf]
