# Supplementary material for: Structural basis for bivalent binding and inhibition of SARS-CoV-2 infection by human potent neutralizing antibodies
Source: Cell Res. 2021 Mar 17;31(5):517–25. doi: 10.1038/s41422-021-00487-9 (PMC7966918; doi:10.1038/s41422-021-00487-9)
Supplement: Supplementary file 14 — Supplementary information, Table S1 [file 41422_2021_487_MOESM14_ESM.pdf]

Supplementary information, Table S1 | Gene family analysis of COVID-19 donor-derived nAbs

mAbs	Heavy chain				Kappa chain (K) or Lambda chain (L)			
	IGHV	HCDR3	HCDR3 length	SHM (%)	IGK(L)V	K(L)CDR3	K(L)CDR 3 length	SHM (%)
P2B-1A1	4-59*01	ARLERDWPLDAFDI	14	0.35	L2-14*01	SSYTSNNTFA	10	1.11
P2B-1A10*	3-53*01	AREGPKSITGTAFDI	15	0.35	K1-33*01,K1D-33*01	QQYDNLPMYT	10	0.38
P5A-3A1	3-53*01	ARDYGDFYFDY	11	0.00	K3-20*01	QQYGSSPRT	9	0.00
P5A-1B8*	3-53*01	ARETLAFDY	9	1.40	K1-9*01	QQLNSYPPA	9	0.00
P5A-2G9*	3-33*01,3-33*06	ARWFHTGGYFDY	12	0.00	L5-37*01	MIWPSNALYV	10	0.35
P5A-1B6	3-30*04,3-30-3*03	ARDGQAITMVQGVIGPPFDY	20	0.00	K1-33*01,K1D-33*01	QQYDNLPYT	9	0.00
P5A-2G7*	4-61*01	ARERCYYGSGRAPRCVWFDP	20	0.34	L2-14*01	SSYTSSSTLVV	11	0.74
P5A-1B9*	4-59*01	ASNGQYYDILTGQPPDYWYFDL	22	0.70	K4-1*01	QQYYSTPLT	9	0.00
P5A-2F11	1-8*01	ARYIVVVPAAKGFDP	15	0.00	K4-1*01	QQYYSTPLT	9	0.00
P5A-3C12*	2-5*02	AHSLFLTVGYSSSWSPFDY	19	0.00	K4-1*01	QQYYSTPHT	9	0.00

The program IMGT/V-QUEST was applied to analyze gene germline, complementarity determining region (CDR) 3 length, and somatic hyper mutation (SHM). The SHM frequency was calculated from the mutated nucleotides.

* Published in the reference (Zhang, et al. Potent and protective IGHV3-53/3-66 public antibodies and their shared escape mutant on the spike of SARS-CoV-2. submitted)
