# Supplementary material for: Structural basis for bivalent binding and inhibition of SARS-CoV-2 infection by human potent neutralizing antibodies
Source: Cell Res. 2021 Mar 17;31(5):517–25. doi: 10.1038/s41422-021-00487-9 (PMC7966918; doi:10.1038/s41422-021-00487-9)
Supplement: Supplementary file 15 — Supplementary information, Table S2 [file 41422_2021_487_MOESM15_ESM.pdf]

Supplementary information, Table S2 | Data collection, 3D reconstruction and model statistic

**Data collection**						
EM equipment	Titan Krios (Thermo Fisher Scientific)					
Voltage (kV)	300					
Detector	Gatan K3 Summit					
Energy filter	Gatan GIF Quantum, 20 eV slit					
Pixel size (Å)	1.087					
Electron dose (e-/Å2)	50					
Defocus range (µm)	-1.2 ~ -2.2					
Number of collected micrographs	2,522	605		2,477	852	
Number of selected micrographs	2,440	566		2,356	796	
Sample	P2B-1A10	P5A-1B8[b]	P5A-1B8[c]	P2A-2G9	P5A-1B6[b]	P5A-1B6[c]
PDB code	7CZQ	7CZR	7CZS	7CZT	7CZU	7CZV
EMDB code	EMD-30513	EMD-30514	EMD-30515	EMD-30516	EMD-30517	EMD-30518
EMDB code (Recenter map, Fig2)		EMD-30871				
**3D Reconstruction**						
Software	cryoSPARC/ Relion					
Number of used particles (whole)	430,062	26,128	16,489	497,874	39,454	55,619
Resolution (whole) (Å)	2.8	3.5	3.6	2.7	3.4	3.3
Number of used particles (local)	860,124	528,018		995,748	161,096	
Resolution (local) (Å)	3.4	3.6		3.0	3.7	
Symmetry	C1					
Map sharpening B factor ($Å^2$)	-90					
**Refinement**						
Software	Phenix					
Cell dimensions (Å)	313.056					
Model composition						
Protein residues	3,868	3,854	4310	3872	3878	4346
Side chains assigned	3,868	3,854	4310	3872	3878	4346
Sugar	70	70	71	70	70	71
R.m.s deviations						
Bonds length (Å)	0.008	0.006	0.006	0.006	0.006	0.006
Bonds Angle (˚)	0.933	0.876	0.877	0.888	0.814	1.858
Ramachandran plot statistics (%)						
Favored	92.74	93.11	90.99	93.79	93.52	92.95
Allowed	7.23	6.86	8.91	6.17	6.45	6.92
Outlier	0.03	0.03	0.10	0.03	0.03	0.13

[a], mono binding; [b], double binding; [c], triple binding**Supplementary information, Table S2, continued**

**Data collection**					
EM equipment	Titan Krios (Thermo Fisher Scientific)				
Voltage (kV)	300				
Detector	Gatan K3 Summit				
Energy filter	Gatan GIF Quantum, 20 eV slit				
Pixel size (Å)	1.087				
Electron dose (e-/Å2)	50				
Defocus range (μm)	-1.2 ~ -2.2				
Number of collected micrographs	892	1,343	2,206	920	
Number of selected micrographs	863	1,282	2,146	897	
Sample	P2B-1A1	P5A-2G7	P5A-1B9	P5A-2F11[b]	P5A-2F11[c]
PDB code	7CZP	7CZW	7CZX	7CZY	7CZZ
EMDB code	EMD-30512	EMD-30519	EMD-30520	EMD-30521	EMD-30522
EMDB code (Recenter map, Fig2)		EMD-30873			
**3D Reconstruction**					
Software	cryoSPARC/ Relion				
Number of used particles (whole)	146,875	211,771	303,289	39,337	55,704
Resolution (whole) (Å)	3.0	2.8	2.8	3.3	3.2
Number of used particles (local)	293,750	137,875	303,289	70,295	
Resolution (local) (Å)	3.4	3.5	3.5	4.2	
Symmetry	C1				
Map sharpening B factor ($Å^2$)	-90				
**Refinement**					
Software	Phenix				
Cell dimensions (Å)	313.056				
Model composition					
Protein residues	3862	3880	4,368	3,880	4349
Side chains assigned	3862	3880	4,368	3,880	4349
Sugar	74	70	71	76	80
R.m.s deviations					
Bonds length (Å)	0.009	0.009	0.005	0.008	0.007
Bonds Angle (˚)	0.994	1.01	0.897	0.907	0.893
Ramachandran plot statistics (%)					
Favored	92.26	91.78	92.44	92.74	92.14
Allowed	7.67	8.19	7.39	7.23	7.69
Outlier	0.07	0.03	0.17	0.03	0.17

[a], mono binding; [b], double binding; [c], triple binding

Supplementary information, Table S2, continued

**Data collection**					
EM equipment	Titan Krios (Thermo Fisher Scientific)				
Voltage (kV)	300				
Detector	Gatan K3 Summit				
Energy filter	Gatan GIF Quantum, 20 eV slit				
Pixel size (Å)	1.087				
Electron dose (e-/Å2)	50				
Defocus range (µm)	-1.2 ~ -2.2				
Number of collected micrographs	1,184	1,071		1,215	1,480
Number of selected micrographs	1,117	958		911	1,401
Sample	P5A-3A1	P5A-3C12[a]	P5A-3C12[b]	P5A-1B8-Fab	P5A-2G7-Fab
PDB code	7D0C	7D0B	7D0D	7D00	7D03
EMDB code	EMD-30530	EMD-30529	EMD-30531	EMD-30523	EMD-30524
EMDB code (Recenter map, Fig2)				EMD-30872	EMD-30874
**3D Reconstruction**					
Software	cryoSPARC/ Relion				
Number of used particles (whole)	74,535	27,776	39,236	145,010	239,537
Resolution (whole) (Å)	3.4	3.9	3.8	3.0	3.2
Number of used particles (local)	149,070	122,049		145,010	95,902
Resolution (local) (Å)	4.2	5.5		4.2	4.0
Symmetry	C1				
Map sharpening B factor ($Å^2$)	-90				
**Refinement**					
Software	Phenix				
Cell dimensions (Å)	313.056				
Model composition					
Protein residues	3860	3206	3468	3854	3412
Side chains assigned	3860	3206	3468	3854	3412
Sugar	70	69	70	70	69
R.m.s deviations					
Bonds length (Å)	0.008	0.006	0.007	0.009	0.009
Bonds Angle (˚)	0.891	0.884	0.876	0.942	0.959
Ramachandran plot statistics (%)					
Favored	92.19	91.25	92.53	92.09	91.66
Allowed	7.71	8.61	7.4	7.84	8.24
Outlier	0.10	0.14	0.07	0.07	0.10

[a], mono binding; [b], double binding; [c], triple binding
