# Supplementary material for: Structural basis for bivalent binding and inhibition of SARS-CoV-2 infection by human potent neutralizing antibodies
Source: Cell Res. 2021 Mar 17;31(5):517–25. doi: 10.1038/s41422-021-00487-9 (PMC7966918; doi:10.1038/s41422-021-00487-9)
Supplement: Supplementary file 16 — Supplementary information, Table S3 [file 41422_2021_487_MOESM16_ESM.pdf]

Supplementary information, Table S3 | Epitopes for all nAbs. Epitope residues involved in ACE2 binding are colored in blue, green or orange for those residues interacting with heavy chain, light chain or both chains of nAb, respectively. The interaction residues are defined by 4 Å distance cut-off.

	G1							G2		G3
ACE2	P2B-1A10	P5A-3A1	P5A-1B8	P5A-2G9	P5A-1B6	P2B-1A1	P5A-2G7	P5A-2F11	P5A-3C12	P5A-1B9
417K	403R	415T	403R	403R	403R	403R	403R	421Y	483V	446G
446G	405D	416G	415T	408R	405D	406E	417K	456F	484E	448N
449Y	406E	417K	416G	409Q	408R	408R	449Y	457R	486F	449Y
453Y	408R	420D	417K	414Q	409Q	409Q	452L	473Y	489Y	452L
455L	415T	421Y	420D	415T	415T	416G	453Y	475A		484E
456F	416G	455L	421Y	416G	416G	417K	455L	476G		485G
475A	417K	456F	455L	417K	417K	449Y	456F	477S		486F
486F	420D	457R	456F	420D	421Y	453Y	484E	484E		487N
487N	421Y	458K	457R	421Y	449Y	455L	485G	485G		489Y
489Y	449Y	460N	460N	446G	453Y	456F	486F	486F		490F
493Q	455L	473Y	473Y	449Y	455L	486F	488C	487N		492L
496G	456F	474Q	475A	456F	456F	487N	489Y	489Y		493Q
498Q	457R	475A	476G	475A	485G	489Y	490F	493Q		494S
500T	458K	476G	486F	476G	486F	493Q	492L			
501N	460N	477S	487N	477S	487N	494S	493Q			
502G	473Y	486F	489Y	486F	489Y	495Y	505Y			
505Y	474Q	487N	493Q	487N	493Q	496G				
	475A	496G	496G	489Y	494S	498Q				
	476G	505Y	500T	493Q	495Y	500T				
	477S		501N	494S	496G	501N				
	487N		502G	496G	501N	502G				
	489Y		505Y	498Q	505Y	505Y				
	493Q			501N						
	494S			505Y						
	496G									
	500T									
	501N									
	502G									
	503V									
	505Y									
